# Transcriptomic Profiles of Rainbow Trout (*Oncorhynchus mykiss*) Selectively Bred for High and Low Fillet Yield

**DOI:** 10.1007/s10126-025-10479-0

**Published:** 2025-06-25

**Authors:** Jamie L. Mankiewicz, Guangtu Gao, Timothy Leeds, Beth M. Cleveland

**Affiliations:** 1 Department of Biological Sciences, North Carolina State University, Raleigh, NC 27695 USA; 2 National Center for Cool and Cold Water Aquaculture, USDA/ARS, Kearneysville, WV 25430 USA

**Keywords:** Fillet yield, RNA-seq, Rainbow trout, Growth, Selective breeding, Aquaculture

## Abstract

The fillet yield phenotype is a trait that can be improved in aquaculture species through conventional selective breeding. This approach was applied to rainbow trout for three consecutive generations of selection to produce a high-yield line (HY) that exhibits 2.5 percentage points higher fillet yield compared to a low-yield line (LY). To characterize the genetic and physiological mechanisms contributing to the HY phenotype, transcriptomic analysis of liver and skeletal muscle was performed at three stages of development, 2 g, 60 g, and 300 g, which corresponded to 35, 208, and 277 days post-hatch. Functional analysis of differentially expressed genes (DEG) suggests that increased muscle yield in the HY line is partially driven by greater hyperplasia at 60 g; although, higher rates of protein accretion, primarily attributed to lower rates of protein degradation, promote muscle cell hypertrophy during all stages of development. Additionally, DEGs support reductions in glycolysis in the HY muscle, with increased activity of the more efficient citric acid cycle and oxidative phosphorylation reactions for energy production compared to the LY line. In the liver, DEGs indicate unique nutrient utilization mechanisms in the HY line that support reduced visceral adiposity compared to the LY line. These findings provide insight into the physiology and metabolism driving the high fillet yield phenotype; this information is useful for the development of genomic markers to enhance breeding strategies toward the improvement of performance traits.

**Supplementary Information:**

The online version contains supplementary material available at 10.1007/s10126-025-10479-0.

## Introduction

Global aquaculture production has rapidly increased, reaching a record high of 122.6 million tons in 2020 (FAO 2022). This growth in aquaculture production must persist to keep up with the demands of animal-source protein supplies for the growing world population (FAO 2022; Boyd et al. 2022). As demand increases for commercial aquaculture production, so does the need for the highest quality farmed fish while maintaining economic sustainability. Approaches to improving growth performance in the aquaculture industry are continually evolving. Some efforts can include altering feed content and feeding diets with different concentrations of lipids and proteins, supplementation, and plant derivatives (Glencross et al. 2007). Another conventional option is the enhancement of economically important production traits through selective breeding. Due to ease of phenotyping and high heritability, selection for faster growing fish is a common trait for improvement through genetic selection in aquaculture (Gjedrem et al. 2012). Selective breeding is valuable for producers to maximize growth in aquaculture, particularly for salmonid breeding programs where there can be a 10 to 15% improvement in growth rate per generation (Leeds et al. 2016; Gjedrem and Thodesen 2005). An increased growth rate that ultimately translates into enhanced feed conversion ratios (FCR) can positively impact productivity; however, the FCR response is generally low and the selection for faster growing and larger fish does not always translate into a greater fillet yield (Vandeputte et al. 2019; Knap and Kause 2018).

The fillet is composed of the primary edible and most valuable portion of the fish, thus greater fillet yield, the ratio of edible fillet to body weight, is desirable in production. Increases in fillet yield can significantly impact profitability yet this trait can be challenging to select upon due to the labor intensive filleting process and inability to analyze the phenotype on live breeding candidates (Vandeputte et al. 2019; Al-Tobasei et al. 2021). Therefore, understanding the genetic and physiological variation contributing to differences in fillet yield can support the development of breeding strategies that improve the efficiency of selection for increased fillet yield.

The USDA National Center for Cool and Cold Water Aquaculture (NCCCWA) has performed genetic selection on rainbow trout (*Oncorhynchus mykiss*) for improved fillet yield over multiple generations (since 2014). This high fillet yield line (HY) has a contemporary low fillet yield line (LY) that was produced from the same founder population after a single generation of downward selection, followed by random breeding thereafter (Cleveland et al. 2024). Although genome-wide association analysis indicates sources of genetic variation contributing to fillet yield phenotypes, physiological mechanisms that differ between the HY and LY lines remain to be characterized (Gonzalez-Pena et al. 2016; Al-Tobasei et al. 2021).

In this study, we utilize a transcriptomics approach to identify biological mechanisms that differ between the HY and LY lines of rainbow trout with divergent fillet yield phenotypes. We target two tissues (the liver and white muscle) during the three key stages of development characterized by unique phases of muscle growth: early rapid growth hyperplasia phase (2 g), hyperplasia/hypertrophy (60 g), and hypertrophy (300 g) (Weatherley et al. 1980). Through the identification of differentially expressed genes (DEG), we aimed to identify biological mechanisms that differ between the lines and contribute to divergent fillet yield phenotypes. These findings are valuable for the development of breeding and husbandry strategies that increase production efficiency in aquaculture.

## Materials and Methods

All procedures and research were approved and performed in accordance with the relevant guidelines and regulations by the Institutional Animal Care and Use Committee at the National Center for Cool and Cold Water Aquaculture (NCCCWA, protocol #098).

### Fish Husbandry

Rainbow trout were reared in tanks supplied with flow-through water (temperature 12.5–13.5 °C, ambient photoperiod) and provided a commercially available feed (Zeigler Finfish G, Gardners, PA, USA; 42% protein, 16% fat) at a fixed percent of tank biomass using automatic feeders (Avro-tec, Huutokoski, Finland). All families for rainbow trout lines were reared separately until fish were approximately 20 g, when they were tagged with passive integrated transponders (PIT) and comingled for grow-out using standard operating procedures for the rearing and husbandry of rainbow trout.

### Experimental Design

This study used two lines of rainbow trout from the NCCCWA selective breeding program. Using fifth-generation families selected for improved growth performance as the founder population (“select” line described in (Leeds et al. 2016)), the high fillet yield line (HY) was selectively bred for increased fillet yield for three generations while the low fillet yield line (LY) was subjected to a single generation of downward selection followed by random breeding for subsequent generations. Six offspring nucleus families representing available genetic diversity were identified from each line that were fertilized the same week and subsequently hatched within a three-day window. Six fish from each family were sampled at three average body sizes: 2 g, 60 g, and 300 g, which corresponded to 35, 208, and 277 days post-hatch (dph). For sampling, fish were euthanized with a lethal dose of MS-222 (300 mg/L). Fork length (FL, mm), body weight (BW, g), and carcass weight (CW, g), which was the body weight of the fish without the head and visceral/gastrointestinal tissues, were recorded. Feed was not provided on the day of sampling. Condition factor (CF) was calculated as (100,000 × g body weight/mm length^3^). Liver and white muscle tissues (*n* = 3 fish per family, *n* = 18 fish per phenotype) were sampled (~ 100 mg of tissue) and placed in 1 mL of RNAlater (Ambion Inc., Austin, TX, USA), kept overnight at 4 °C, and then stored at − 80 °C until extractions. White muscle samples were extracted from epaxial muscle regions ventral to the dorsal fin. Percent carcass yield was calculated as CW × BW^−1^ × 100. Due to their small size, only BW was recorded for fish harvested at the 2 g sampling.

### Sample Processing, RNA Isolation, and Quantitative Real-Time PCR (qPCR)

Total RNA was extracted and purified from tissues with 1 mL of Tri-Reagent (Molecular Research Center, Cincinnati, OH, USA) and Direct-zol miniprep spin columns with DNase treatment using standard methods from the manufacturer (Zymo Research, Irvine, CA). RNA was initially quantified by the absorbance OD 260:280 ratio using a Nanodrop 2000c spectrophotometer (Thermo Scientific, Waltham, MA, USA). Quality and quantity of RNA were more accurately assessed using the Agilent Bioanalyzer 2100 (Santa Clara, CA, USA) prior to sending samples for RNA-seq analysis and ranged 1.99–1.89 for muscle and 1.96–1.80 for liver samples. Equal amounts of RNA from three individual fish within each HY or LY family were pooled for RNA sequencing. There were, however, six liver samples that were pools of only two RNA samples, due to low RNA quality of the third sample. Samples that were sent for RNA-seq exhibited an average RIN value of 9.3; all were above a RIN of 8.0. Sequencing services were performed by GENEWIZ (South Plainfield, NJ, USA) and included individual library preparation and sequencing on the Illumina HiSeq platform with 2 × 150 base pair configuration (~ 350 million reads/lane).

RNA-seq data were validated via quantitative polymerase chain reaction (qPCR). All samples were diluted, and 1 µg of total RNA was used in a cDNA synthesis reaction via reverse transcription following the manufacturer’s instructions (Promega M-MLV Reverse Transcriptase, Madison, WI, USA). Genes that were metabolically relevant and common across the different sampling body sizes were assessed when possible. In the muscle, levels of mRNA were measured for *hspg2*, heparan sulfate proteoglycan 2; *pfkmb*, phosphofructokinase; *col22a1*, collagen type XXII alpha 1 chain; *gapdh*, glyceraldehyde-3-phosphate dehydrogenase; *igf-1*, insulin-like growth factor 1. In the liver, mRNA levels of *mfap4*, microfibril associated protein 4; *fabp2*, fatty acid-binding protein 2; *col18a1*, collagen type XVIII alpha 1 chain; *pebp1*, phosphatidylethanolamine-binding protein 1; and *insr2*, insulin receptor 2 were determined by qPCR using gene-specific primers (Supplementary File 1). Primer pairs were designed with Primer-3 and BLAST on NCBI (Untergasser et al. 2012). Primers designed for genes with other paralogs were compared to each other using BLAST to ensure no sequence complementation.

All reactions were run in triplicate and performed on a QuantStudio 5 Real-Time PCR System (Applied Biosystems, Foster City, CA, USA), with Applied Biosystems SYBR Green qPCR master mix, using 1.5 µM primers and 2 µL of 1:6 diluted cDNA in a total reaction volume of 10 µL. The cycling parameters were 95 °C for 10 min, followed by 40 cycles of 95 °C for 30 s, and 60 °C for 1 min. A dissociation melt curve step at the end was performed to verify a single PCR product. Negative controls included using water instead of RNA template (no template control; NTC) and DNase-treated RNA with no reverse transcriptase enzyme to ensure no genomic DNA contamination (no amplification control; NAC). Cycle threshold (Ct) values for samples were transformed using a standard curve of serially diluted pooled cDNA versus Ct values (*R*^2^ = 0.992–0.999). Samples were then normalized to reflect the amount of template cDNA per ng total RNA (cDNA/ng total RNA) loaded into each reaction (Huggett et al. 2005; Bustin 2000). The values are expressed as fold change relative to the mean of the HY group of the same body size and tissue.

### RNA-seq Data Analysis

For all RNA-seq reads, the Illumina universal adaptors were trimmed, and reads that were shorter than 60 bases after the adaptor trimming were removed with the program cutadapt (version 2) (Martin 2011). After trimming and removing short reads, 99% of the reads were retained. The sequences were then aligned to the rainbow trout genome (Gao et al. 2021) (Omyk_1.0, GCF_.002163495.1 and USDA_OmykA_1.1, GCF_013265735.2) using a splicing aware sequence aligner, STAR (version 2) (Dobin et al. 2013). In building the STAR reference library, intron junction sites in the genome were marked using the RefSeq annotation data. Read pairs mapped to the genes were counted using htseq-count (Version 0.12) (Anders et al. 2015). Reads that mapped to multiple locations were excluded. The raw count data were processed with the R package, DESeq2 (Version 1) (Love et al. 2014), to test for differential expression of the genes in HY line compared with the LY line in both liver and white muscle samples at each body size (2 g, 60 g, and 300 g). In each comparison, biological replicates from six pooled samples were used for both phenotypes, for each liver and muscle at the three different body sizes. Differentially expressed genes (DEGs) were selected based on the adjusted *P*-value (padj) calculated with the Wald test (to generate *P*-values) followed by the FDR/Benjamini–Hochberg correction (to generate adjusted *P*-values). Genes with padj ≤ 0.2 (uncorrected *p*-value ≤ 0.01) were subject to a one-way analysis of variance (ANOVA) using PC-SAS (version 9.2, Cary, NC, USA); those exhibiting a main effect of line (*P* < 0.05) were considered DEGs. ANOVA analysis was performed on normalized count values to identify DEGs that fell outside the strict parameters of padj < 0.05 yet were of physiological significance. The value of this approach is the identification of a greater number of DEGs, often with more moderate levels of regulation (0.5 < fold change < 1.5), to advance the detection of significantly enriched pathways. The HY and LY lines differ in fillet yield by only a few percentage points, which is a relatively small but economically important difference. Given that fillet yield is a multigenic trait, regulated by many genes, we hypothesize that genetic variation stemming from selective breeding for this trait had moderate effects on multiple physiological pathways rather than large effects on just a few. The relaxed stringency will support the detection of these pathways, although the caveat is the increased likelihood that DEGs are “false positives,” identified as such simply due to chance. Placing emphasis on physiological significance of enriched pathways and functions, rather than specific DEGs as biomarkers, can minimize the chance of over-stating the impact of DEGs that are simply false positives.

Identification of canonical pathways and biological functions enriched by DEGs was performed using fold-change values in Ingenuity Pathway Analysis (IPA; QIAGEN, Hilden, Germany). For pathway enrichment analysis, *P*-values and *z*-scores were determined by IPA software. Human gene orthologs were used to stand for rainbow trout DEGs for enrichment analyses and were identified using the Aquamine database (https://aquamine.elsiklab.missouri.edu/) and by searching on GenBank on NCBI (National Center for Biotechnology Information, Bethesda, MD, USA; NCBI 1988). Due to the two rounds of genome duplication (teleost specific and salmonid specific duplications), in the rainbow trout, there can be up to four gene paralogs corresponding to the same human gene ortholog; thus, potentially, up to four DEGs share the same human gene symbol. Because the IPA software will only accept a single-DEG value per gene symbol, in the event that two or more gene paralogs were differentially expressed, the mean of the fold-change value was used for functional analysis. In 75% of these cases, the directional change in gene expression was shared by all paralogs. Heat maps for DEGs were generated using Heatmapper with the normalized count data, using average linkage and the Pearson method of distance measurement (Babicki et al. 2016). Raw sequence data is archived in NCBI as BioProject number PRJNA1242221 (https://www.ncbi.nlm.nih.gov/bioproject/).

### Statistical Analyses

As previously stated, for RNA-seq, the genes with Padj ≤ 0.2 (uncorrected *P*-value ≤ 0.01) were analyzed by one-way ANOVA using PC-SAS (version 9.2, Cary, NC, USA), and those samples with *P* < 0.05 were characterized as DEGs. The main effects of genetic line on morphology traits were detected by one-way ANOVA. A simple linear regression was used for qPCR data. These analyses were performed using GraphPad Prism 8 (GraphPad, La Jolla, CA, USA). The level set for statistical significance for all analyses was *P* < 0.05, and data are shown as mean values ± SEM.

## Results

### Sampling and Growth Data for HY and LY Lines

**Fig. 1:**
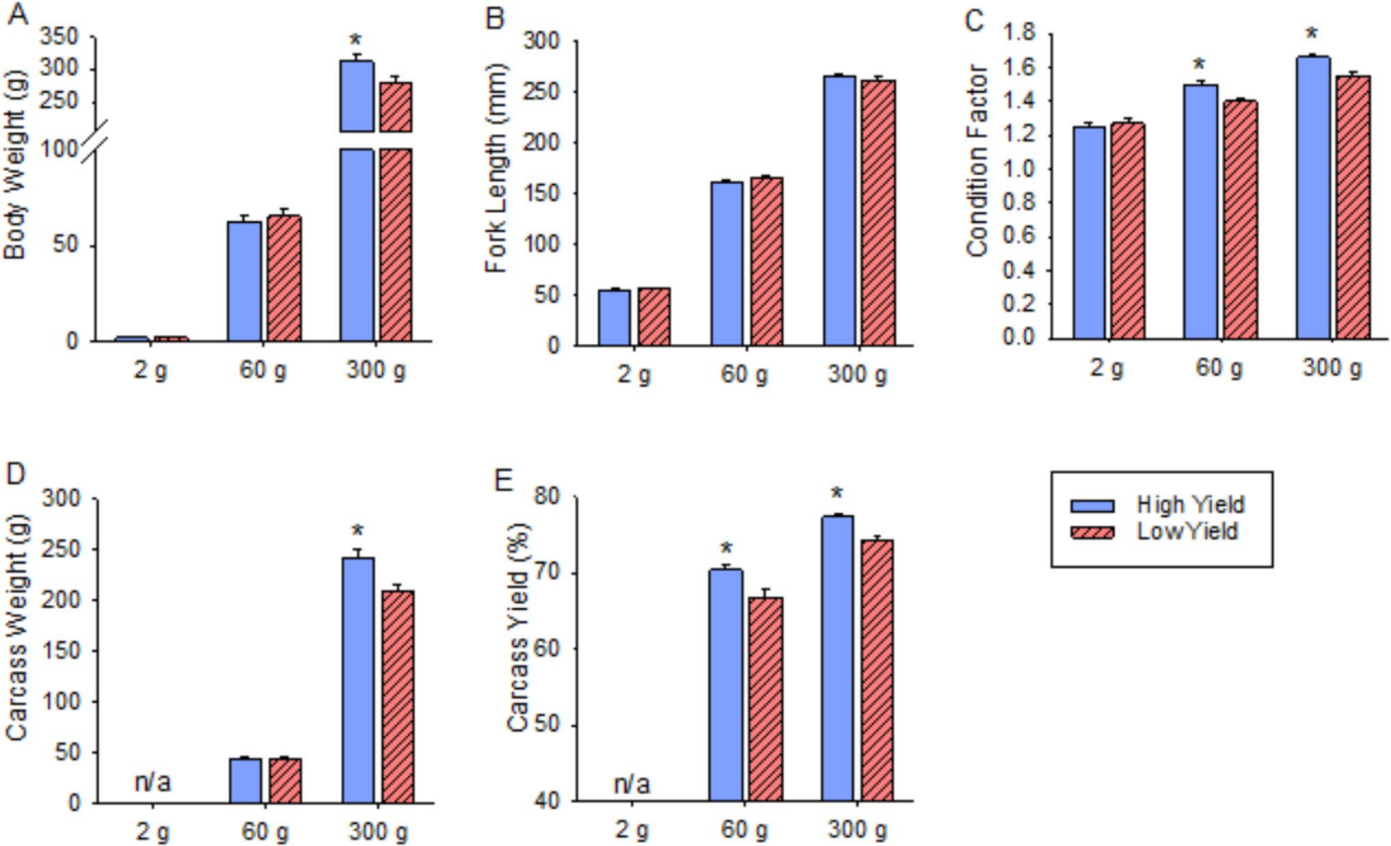
Body morphometrics and fillet yield metrics in the high-yield and low-yield lines. Mean body weight (**A**), fork length (**B**), condition factor (**C**), absolute carcass weight (**D**) and fractional carcass weight (**E**) are shown. Asterisks indicate a difference between the lines within the same size (*P* < 0.05). Bars indicate means ± SEM

The mean values for body morphology variables are presented in Fig. 1. Target BW at sampling was 2 g, 60 g, and 300 g; the actual mean BW for the HY line was 2.1 ± 0.1 g, 62.7 ± 3.4 g, and 312.4 ± 12.0 g, and the LY line was 2.4 ± 0.1 g, 65.2 ± 3.5 g, and 280.8 ± 9.4 g. The HY fish were significantly lighter and heavier at the 2 g and 300 g sampling, respectively (*P* < 0.05). Fork length did not differ between HY and LY fish. Condition factor was significantly greater in HY fish at the 60 g (HY, 1.50 ± 0.02; LY, 1.40 ± 0.02; *P* < 0.001) and 300 g (HY, 1.66 ± 0.02; LY, 1.55 ± 0.02; *P* < 0.001) samplings but not at the 2 g sampling (HY, 1.25 ± 0.02; LY, 1.27 ± 0.03). Absolute CW (HY, 44.3 ± 2.5 g; LY, 44.3 ± 2.6 g; *P* > 0.99) did not show differences between lines at the 60 g sampling; however, by the 300 g sampling, the HY fish exhibited significantly greater CW (HY, 241.8 ± 9.5 g; LY, 208.7 ± 6.9 g; *P* = 0.003). The percent carcass yield was significantly greater in the HY line at both the 60 g (HY, 70.4 ± 0.8; LY, 66.9 ± 0.9; *P* = 0.003) and 300 g samplings (HY, 77.4 ± 0.5; LY, 74.4 ± 0.6; *P* < 0.001; Fig. 1).

### RNA-seq Alignments

A total of 72 samples were sequenced from two genetic lines (six different families of each fillet yield, HY and LY, *n* = 36 per line) at three average body sizes, 2 g, 60 g, and 300 g. A summary of total RNA-seq reads generated for each sample after the adaptor filtering, and the corresponding STAR alignment results can be found in supplementary files (Supplementary File 2). RNA-seq reads submitted for the alignment for each sample range from approximately 20 to 65 million pairs. On average, 78.8% of the read pairs were uniquely mapped to the reference genome, and 90.8% of the uniquely mapped read pairs were used by htseq-count as the qualified mappings for the count table at each gene.

### Differentially Expressed Genes Between HY and LY Lines

**Fig. 2:**
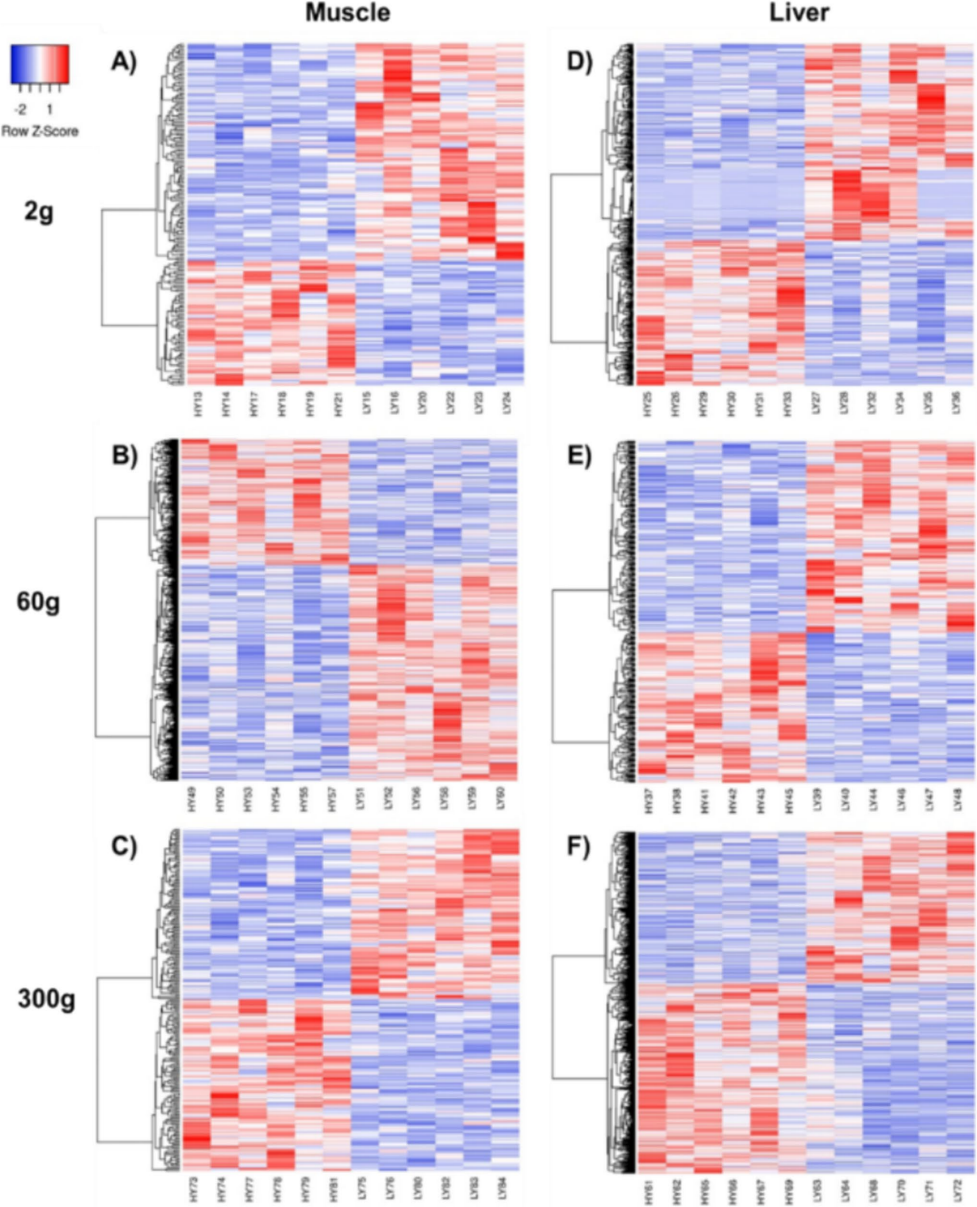
Heat maps of differentially expressed genes (DEGs) from the transcriptomic comparison of high fillet yield (HY) and low fillet yield (LY) rainbow trout in the white muscle (**A**–**C**) and liver (**D**–**F**). Blue boxes represent downregulated DEGs, while red boxes represent upregulated DEGs. Samples were analyzed at different body sizes, 2 g, 60 g, and 300 g average weight

**Fig. 3:**
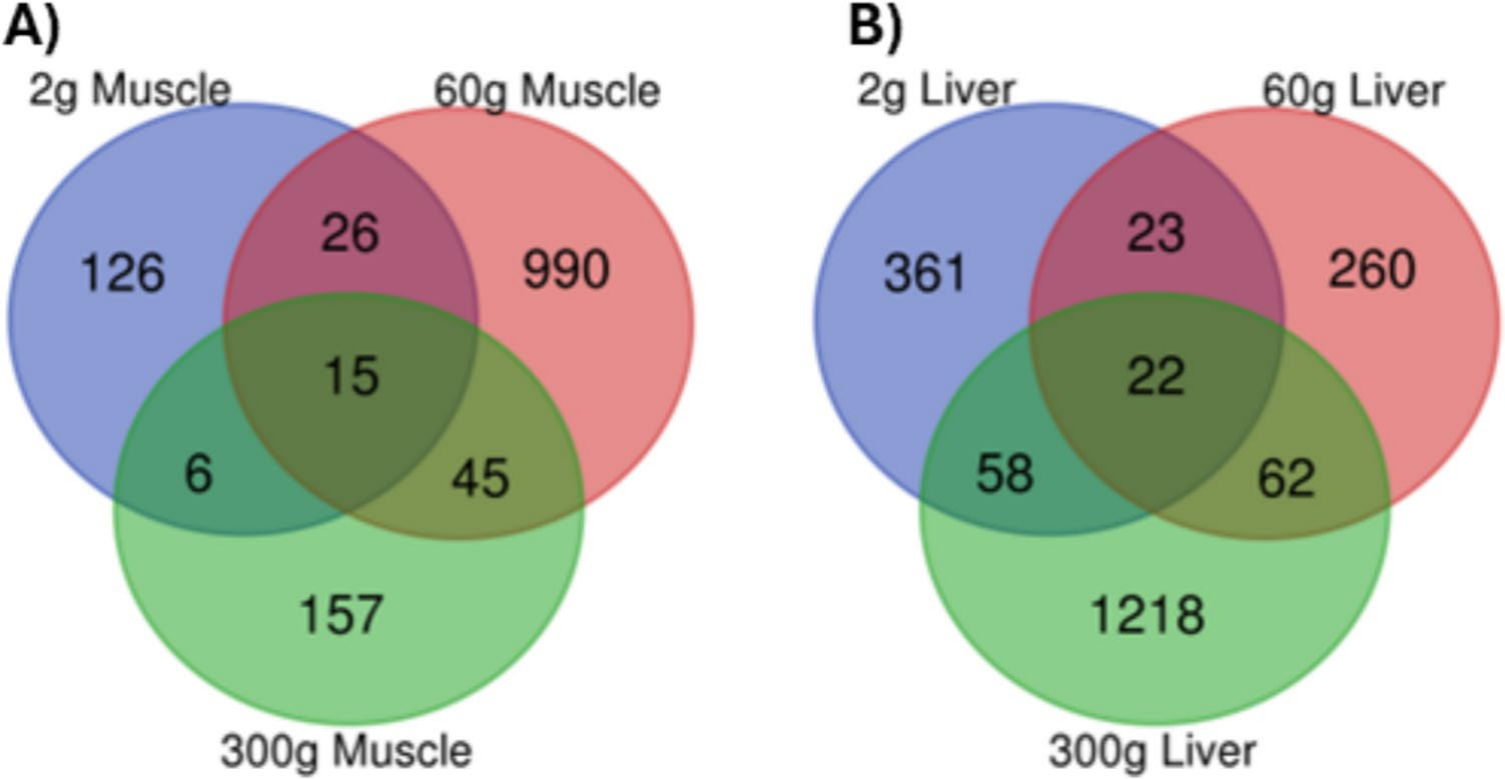
Venn diagrams showing an overlap in unique differentially expressed genes (DEGs) from the transcriptomic comparison of high fillet yield (HY) and low fillet yield (LY) rainbow trout at different sizes, 2 g (blue), 60 g (red), and 300 g (green) average body weight. Samples were analyzed in **A** white muscle and **B** liver

The total number of DEGs were 3,663 between HY and LY samples at all body sizes and tissue types. There were 173, 1076, and 223 DEGs in the muscle tissue and 464, 367, and 1360 DEGs in the liver tissue for 2 g, 60 g, and 300 g fish, respectively (Supplementary File 3). Overall, there was a greater number of DEGs in liver (2191 DEGs), pooled across all samplings, compared to muscle tissue (1472 DEGs). Heatmaps visually show the up- and downregulated DEGs for each family within the sampling size and the tissue between the HY and LY lines (Fig. 2). The Venn diagram in Fig. 3 indicates overlap in unique DEGs for each tissue at different samplings. For the muscle, only 15 DEG (14 protein coding, 1 lncRNA) were shared by fish at the sampling points. There was also low DEG overlap between sampling points in the liver; 22 protein coding DEGs were common across all sizes. On average, 90.7% of the DEGs mapped to annotated proteins (range, 86–95%) with 150, 1024, and 199 DEGs in muscle and 418, 330, and 1263 DEGs in liver that mapped to human protein coding genes. Ultimately, unique DEG symbols used for IPA analysis totaled 139, 912, and 199 in muscle and 387, 321, and 1159 in liver at the 2 g, 60 g, and 300 g sampling, respectively.

### Pathway Enrichment Analysis: Muscle

**Fig. 4:**
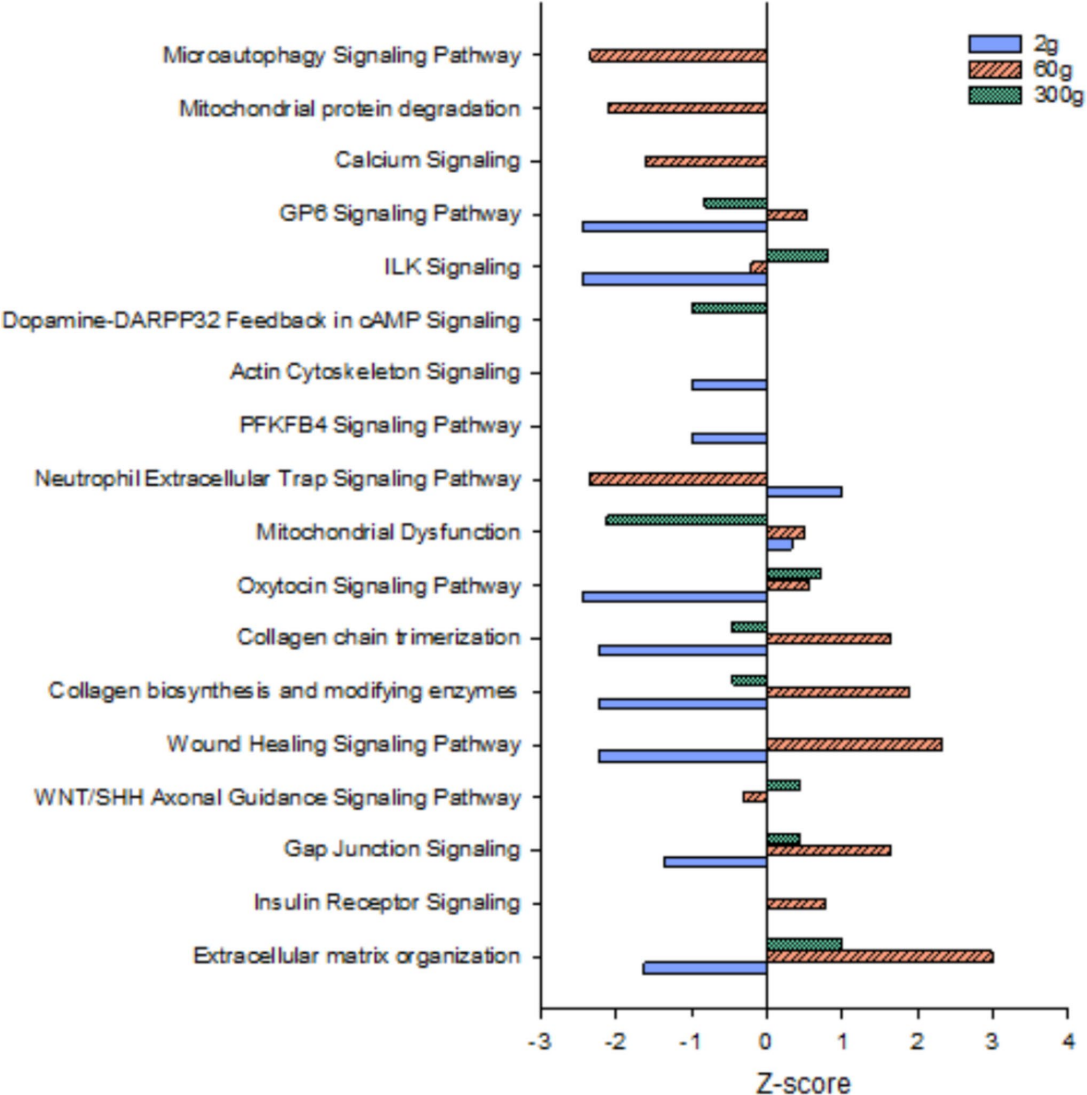
Overlapping enriched pathways (*P* < 0.05) in muscle tissue of high fillet yield vs. low fillet yield rainbow trout at three different sizes, 2 g, 60 g, and 300 g average body weight. *Z*-scores indicate the overall direction of pathway regulation

The top enriched pathways for each body size, along with the *P*-values, *z*-scores, percent enrichment, and number of DEGs, are presented for the muscle at each sampling size in Table 1. Despite only sharing 15 DEGs across all sampling sizes, there were a number of common pathways that were enriched with DEGs (Fig. 4). However, there were no common pathways regulated in the same direction for each sampling point, suggesting that, within muscle, unique metabolic and mitogenic mechanisms contribute to the HY phenotype at different growth phases. Additionally, numerous pathways exhibited a *z*-score of zero (no bar shown), indicating the pattern of DEG expression does not support pathway upregulation (positive *z*-score) or downregulation (negative *z*-score). In general, pathways that supported the organization of extracellular matrix/collagen organization were downregulated in HY fish at the 2 g size, but upregulated at 60 g. Signaling pathways associated with the extracellular matrix also tended to be downregulated in the HY line at the 2 g size (wound healing signaling, gap junction signaling, actin cytoskeleton signaling, ILK signaling). In contrast, several of these pathways were upregulated in the HY line at the 60 g size (gap junction signaling, wound healing signaling), along with three unique pathways downregulated in the HY line only at this stage (microautophagy, mitochondrial protein degradation, calcium signaling). At the 300 g body weight, the HY line was unique regarding the upregulation of ILK signaling and the downregulation of dopamine feedback in cAMP signaling.

**Table 1:** Top enriched pathways in muscle tissue of high fillet yield vs. low fillet yield rainbow trout

Body size	Top muscle pathway	*P*-value	BH *P*-value	*Z*-score	% enrichment	# of DEG
2 g	Striated muscle contraction	6.38E − 10	3.85E − 07	− 1.89	19.4	7
	Collagen chain trimerization	3.22E − 06	6.48E − 04	− 2.24	11.3	5
	GP6 signaling pathway	5.64E − 05	2.83E − 03	− 2.45	4.7	6
	Mitochondrial dysfunction	8.27E − 05	3.56E − 03	0.33	2.6	9
	White adipose tissue browning pathway	8.92E − 05	3.66E − 03	− 1.34	4.3	6
	CLEAR signaling pathway	1.88E − 05	1.26E − 03	− 2.12	3.2	9
	PFKFB4 signaling pathway	1.23E − 04	4.62E − 03	− 1.00	8.2	4
	ILK signaling	5.84E − 04	2.14 E − 02	− 2.45	3.1	6
	FGF signaling	1.06E − 03	2.21E − 02	− 1.00	4.7	4
	Protein kinase A signaling	1.11E − 03	2.55E − 02	− 0.45	2.0	8
	EIF2 signaling	1.55E − 03	3.01E − 02	0.45	2.5	6
	Docosahexaenoic acid (DHA) signaling	2.03E − 03	3.33E − 02	− 1.63	2.4	6
60 g	Glycolysis I	7.25E − 11	7.40E − 08	− 2.11	41.3	12
	Response of EIF2AK4 (GCN2) to amino acid deficiency	2.77E − 10	1.41E − 07	− 3.13	19.4	20
	Striated muscle contraction	1.40E − 09	4.77E − 07	− 2.31	33.3	12
	Calcium signaling	2.00E − 09	5.09E − 07	− 1.60	12.8	28
	Signaling by Rho family GTPases	3.34E − 09	5.80E − 07	0.20	11.6	31
	Mitochondrial dysfunction	3.41E − 09	5.80E − 07	0.51	10.4	36
	ILK signaling	4.19E − 09	6.11E − 07	− 0.63	13.2	26
	EIF2 signaling	5.54E − 08	4.03E − 06	− 2.32	11.3	27
	Gluconeogenesis I	4.62E − 07	2.14E − 05	− 1.41	30.0	9
	IGF-1 signaling	5.60E − 07	2.38E − 05	− 0.26	15.2	16
	Serotonin receptor signaling	1.03E − 06	3.85E − 05	1.33	8.1	38
	mTOR signaling	1.36E − 06	4.63E − 05	0.28	10.8	23
300 g	2-Ketoglutarate dehydrogenase complex	1.36E − 06	8.88E − 04	n/a	75.0	3
	Collogen chain trimerization	1.42E − 05	4.62E − 03	− 0.45	11.4	5
	GP6 signaling pathway	3.10E − 04	4.03E − 02	− 0.82	4.7	6
	ILK signaling	5.23E − 04	4.40E − 02	0.82	3.5	7
	Fatty Acyl-CoA biosynthesis	2.23E − 03	1.12E − 01	n/a	8.1	3
	Lysine catabolism	3.10E − 03	1.23E − 01	n/a	16.7	2
	Mitochondrial dysfunction	3.18E − 03	1.25E − 01	− 2.12	2.3	8
	Glucose metabolism	3.21E − 03	1.28E − 01	− 2.00	4.7	4
	p53 signaling	5.31E − 03	1.43E − 01	n/a	4.0	4
	Microautophagy signaling pathway	5.42E − 03	1.47E − 01	n/a	3.1	5
	TCA cycle II	1.02E − 02	1.93E − 01	n/a	8.7	2
	Complex III assembly	1.08E − 02	2.11E − 01	n/a	8.0	2

Enrichment *P*-values and Benjamini–Hochberg (BH) corrected *P*-values are shown. *Z*-scores indicate the overall direction of pathway regulation. The percent enrichment indicates the number of DEG compared to the total number of pathway genes. The number of DEG within each pathway is also indicated

**Fig. 5:**
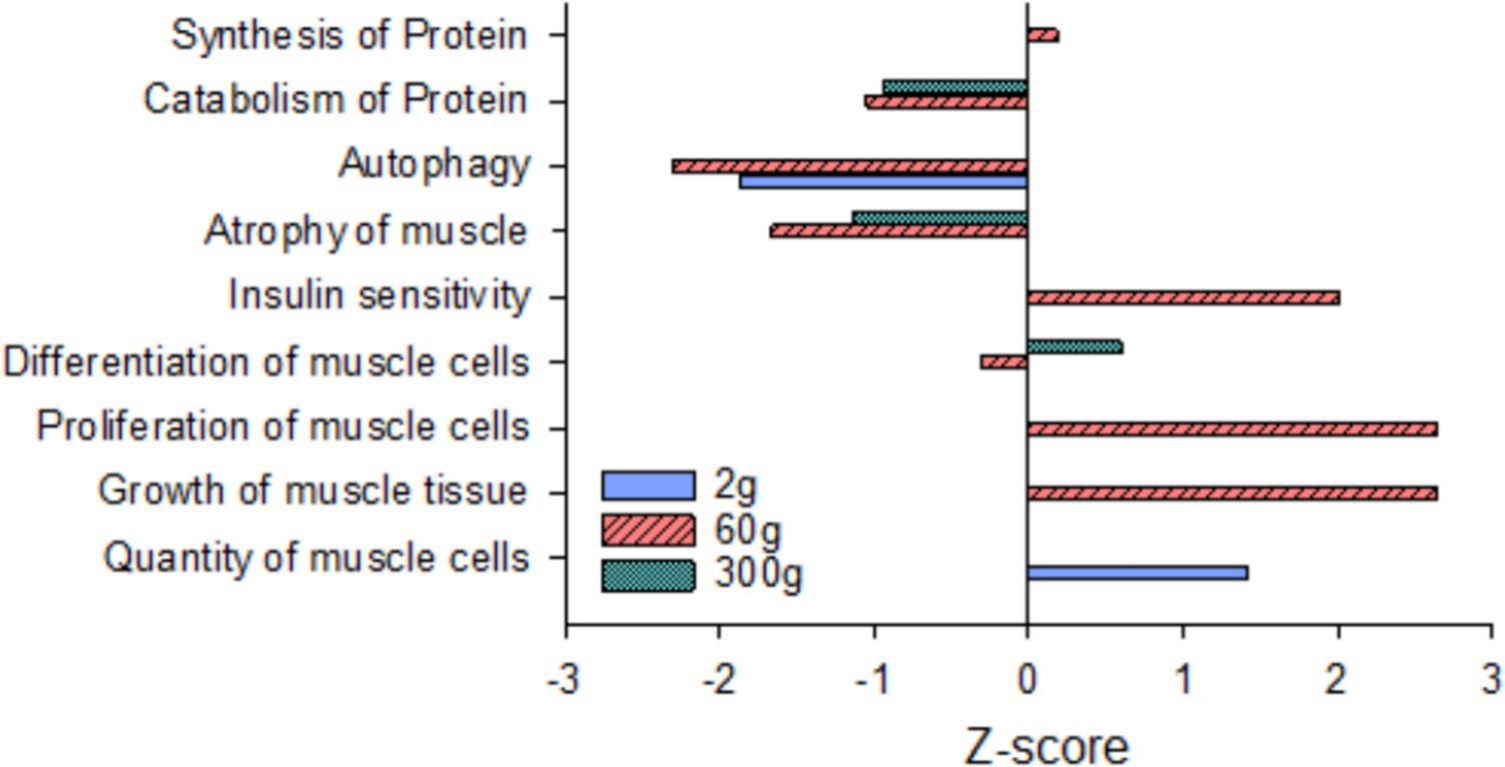
Enrichment of biological functions related to muscle growth in muscle tissue of high fillet yield vs. low fillet yield rainbow trout at three different sizes, 2 g, 60 g, and 300 g average body weight. *Z*-scores indicate the overall direction of pathway regulation

Biological processes directing myogenesis and muscle growth were evaluated for DEG enrichment at each body weight (Fig. 5). At the 2 g size was enrichment of DEG associated with “quantity of muscle cells” (*P* = 0.003), suggesting that enhanced myogenic processes in the HY line are initiated prior to the 2 g sampling. DEGs associated with “proliferation of muscle cells” (*P* = 1.14E − 05) support upregulation of hyperplasia at the 60 g sampling, coupled with down regulation of “differentiation of muscle cells” (*P* = 1.17E − 10). DEGs also reflect enhanced “insulin sensitivity” (*P* = 1.45E − 05) in the HY line at the 60 g sampling, pointing to a possible signaling mechanism directing these responses. Consistent across all sizes is a reduced capacity for proteolytic process in the HY line, including “atrophy of muscle” at both 60 g (*P* = 2.80E − 08) and 300 g (*P* = 0.001), “autophagy” at 2 g (*P* = 0.006) and 60 g (*P* = 3.43E − 12), and “catabolism of protein” at 60 g (1.07E − 13) and 300 g (*P* = 0.004). Additionally, DEGs associated with “synthesis of protein” were upregulated in the HY line at the 60 g size (*P* = 5.91E-12).

#### Pathway Analysis: Liver

**Fig. 6:**
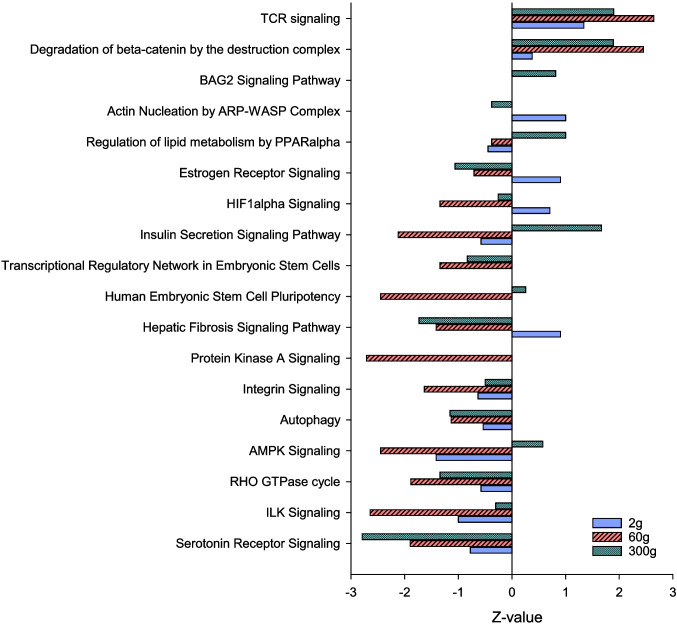
Overlapped enriched pathways (*P* < 0.05) in liver tissue of high fillet yield vs. low fillet yield rainbow trout at three different sizes, 2 g, 60 g, and 300 g average body weight. *Z*-scores indicate the overall direction of pathway regulation

The top enriched pathways for each body size, along with the *P*-values, *z*-scores, percent enrichment, and number of DEGs, are presented for the liver at each sampling size in Table 2. Unlike the muscle, in the liver, enriched pathways (*P* < 05) that were shared among all three sampling sizes exhibited a higher tendency for regulation in the same direction (Fig. 6). For example, regardless of age, “TCR signaling” and “the degradation of beta-catenin by the destruction complex” had positive *z*-scores characteristic of upregulation. In contrast, pathways associated with “integrin signaling,” “autophagy,” “RHO GTPase cycle,” “ILK signaling,” and “serotonin receptor signaling” were downregulated with negative *z*-scores. Also notable was a downregulation of “insulin secretion signaling pathway” in the HY line at 2 g and 60 g, while this pathway was upregulated at 300 g. “Protein kinase A signaling” also exhibited a negative *z*-score in the HY line at 60 g.

**Table 2:** Top enriched pathways in liver tissue of high fillet yield vs. low fillet yield rainbow trout

Body size	Top muscle pathway	*P*-value	BH *P*-value	*Z*-score	% enrichment	# of DEG
2 g	SPINK1 pancreatic cancer pathway	2.19E − 09	1.83E − 06	2.24	16.9	11
	Autophagy	4.08E − 06	1.71E − 03	− 0.54	6.3	14
	Cellular response to heat stress	1.10E − 04	2.21E − 02	1.89	7.9	8
	Docosahexaenoic acid (DHA) signaling	3.15E − 04	3.10E − 02	− 0.91	4.7	12
	ERB2-ERBB3 signaling	3.22E − 04	3.32E − 02	0.82	9.3	6
	ERK/MAPK signaling	3.81E − 04	3.38E − 02	− 0.91	5.0	11
	VEGF family ligand-receptor interactions	5.21E − 04	3.47E − 02	0.45	7.2	7
	Insulin secretion signaling pathway	8.05E − 04	3.71E − 02	− 0.58	4.3	12
	IL-7 signaling pathway	9.96E − 04	3.74E − 02	− 0.45	7.5	6
	Tryptophan catabolism	9.65E − 04	3.85E − 02	n/a	21.4	3
	mTOR signaling	1.16E − 03	4.56E − 02	0.33	4.6	10
	Growth hormone signaling	4.11E − 03	7.21E − 02	1.00	6.8	5
60 g	Cellular response to hypoxia	3.81E − 06	1.85E − 03	2.83	10.5	8
	RAR activation	2.36E − 05	4.85E − 03	− 2.67	3.9	17
	Protein ubiquitination pathway	3.85E − 05	6.33E − 03	1.51	4.6	13
	Metabolism of polyamines	7.75E − 05	9.18E − 03	2.45	10.1	6
	Regulation of lipid metabolism by PPARalpha	6.17E − 04	3.15E − 02	− 0.38	5.8	7
	Tryptophan degradation to 2-amino-3-carboxymuconate Semialdehyde	2.11E − 03	4.21E − 02	n/a	33.3	2
	Maturity onset diabetes of Young (MODY) Signaling	2.55E − 03	0.48E − 02	n/a	6.4	5
	ILK signaling	2.81E − 03	0.55E − 02	− 2.65	4.0	8
	Senescence pathway	3.79E − 03	1.58E − 02	− 1.89	3.3	10
	Autophagy	5.31E − 03	0.81E − 02	− 1.13	3.6	8
	Insulin secretion signaling pathway	7.09E − 03	0.89E − 02	− 2.12	3.2	9
300 g	EIF2 signaling	1.65E − 15	9.07E − 13	1.71	18.0	43
	tRNA charging	3.95E − 15	1.45E − 12	4.24	47.3	18
	Regulation of eIF4 and p70S6K signaling	1.88E − 07	1.47E − 05	n/a	13.8	26
	Insulin secretion signaling pathway	6.14E − 07	4.21E − 05	1.67	11.4	32
	Estrogen receptor signaling	4.70E − 06	2.35E − 04	− 1.06	9.4	39
	Aryl hydrocarbon receptor signaling	4.95E − 06	2.37E − 04	− 1.29	12.5	23
	Sumoylation pathway	7.20E − 06	3.16E − 04	− 0.83	15.8	16
	Serotonin receptor signaling	4.49E − 05	1.76E − 03	− 2.79	8.5	40
	GADD45 signaling	4.81E − 05	1.82E − 03	− 0.30	18.3	11
	Regulation of lipid metabolism by PPARalpha	5.83E − 05	2.07E − 03	1.00	13.4	16
	γ-linolenate biosynthesis II (animals)	1.11E − 04	3.48E − 03	− 1.63	31.5	6
	Cellular response to heat stress	1.19E − 04	3.64E − 03	1.51	13.8	14

Enrichment *P*-values and Benjamini–Hochberg (BH)-corrected *P*-values are shown. *Z*-scores indicate the overall direction of pathway regulation. The percent enrichment indicates the number of DEG compared to the total number of pathway genes. The number of DEG within each pathway is also indicated

**Fig. 7:**
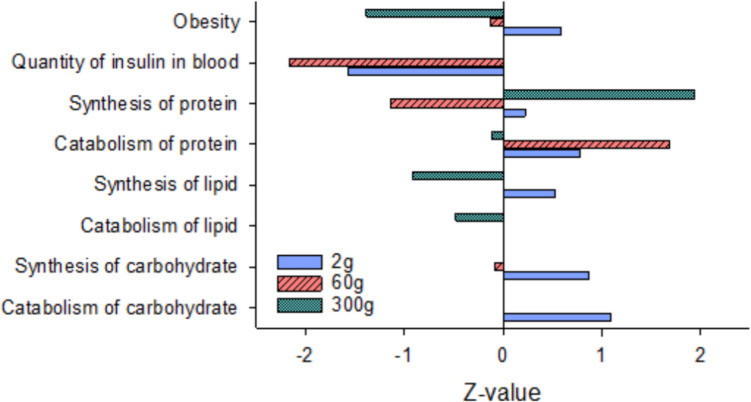
Enrichment of metabolic processes directing nutrient partitioning in liver tissue of high fillet yield vs. low fillet yield rainbow trout at three different sizes, 2 g, 60 g, and 300 g average body weight. *Z*-scores indicate the overall direction of pathway regulation

Biological processes in the liver that direct nutrient utilization and partitioning were investigated for DEG enrichment at each sampling point (Fig. 7). Findings support that the HY line exhibits greater reliance on carbohydrate metabolism for energy production at smaller sizes, given the positive *z*-scores associated with “synthesis of carbohydrate” (*P* = 0.001) and “catabolism of carbohydrate” (*P* = 0.0014) in 2 g fish. DEGs also reflect reduction in the “quantity of insulin in blood” in the HY line at both 2 g (*P* = 0.005) and 60 g (*P* = 3.59E-04), suggesting that peripheral tissues are more sensitive to insulin signaling (consistent with this enriched pathway in 60 g muscle). In contrast, at the larger 300 g size, the HY line may exhibit lower reliance on lipids for energy, as the metabolic processes of “synthesis of lipid” (*P* = 8.89E − 09) and “catabolism of lipid” (*P* = 8.19E − 06) were enriched and downregulated. Mechanisms of protein turnover were also enriched in the HY line, with an upregulation of DEGs associated with “catabolism of protein” at 2 g (*P* = 1.10E − 06) and 60 g (*P* = 0.0031), although “synthesis of protein” was downregulated at 60 g (*P* = 0.0066) and upregulated at 2 g (*P* = 9.11E − 05) and 300 g (*P* = 2.10E − 15). DEGs also suggested reduced “obesity” (60 g, *P* = 3.07E − 04; 300 g, *P* = 5.24E − 05) in the HY line at the two heavier body weights.

### Network Analysis

**Fig. 8:**
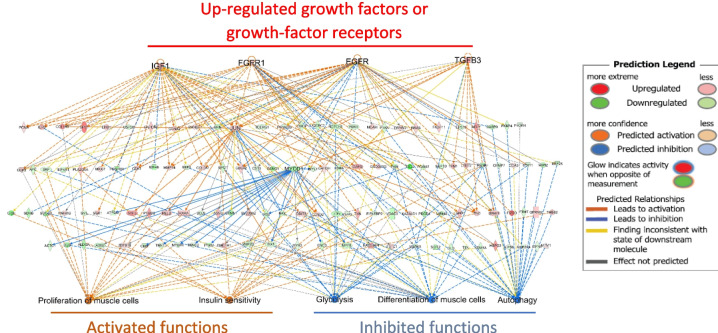
Pathways supporting increased muscle growth in the high yield (HY) line. Connections between upregulated growth factors or growth factor receptors, differentially regulated genes (DEGs), and predicted changes in biological functions that support greater muscle growth in the HY line (60 g sampling)

The higher percent carcass yield of the HY line first occurred at the 60 g sampling, indicating that physiological mechanisms during this period of development are significant for the HY phenotype. Transcripts for several growth factors or growth factor receptors were elevated in the HY line (*igf1*, *egfr*, *tgfb3*, *fgfr1*), suggesting increased capacity for anabolic signaling in white muscle. Relationships between these growth factors or growth factor receptors, DEGs, and functional outcomes, generated using the IPA database of known and predicted relationships (derived primarily from mammalian studies), are shown in Fig. 8. Increased activity of growth factor signaling pathways is generally supported by complementary changes in DEGs that are associated or directly regulate the functions of muscle cell proliferation or differentiation, insulin sensitivity, glycolysis, and autophagy (*P* < 0.05). One of the central signaling pathways downstream of all growth factors involves the Jun protein, which was also a DEG upregulated in the HY line identified as an “activated upstream molecule” potentially driving differential changes in the dataset (*P*-value = 0.01, *z*-score = 2.80). Collectively, increased signaling through growth factor responsive pathways is predicted to regulate biological processes that stimulate muscle growth and contribute to the HY phenotype, specifically activating proliferation of muscle cells and insulin sensitivity, and inhibiting glycolysis, differentiation of muscle cells, and autophagy (*P* < 0.5).

**Fig. 9:**
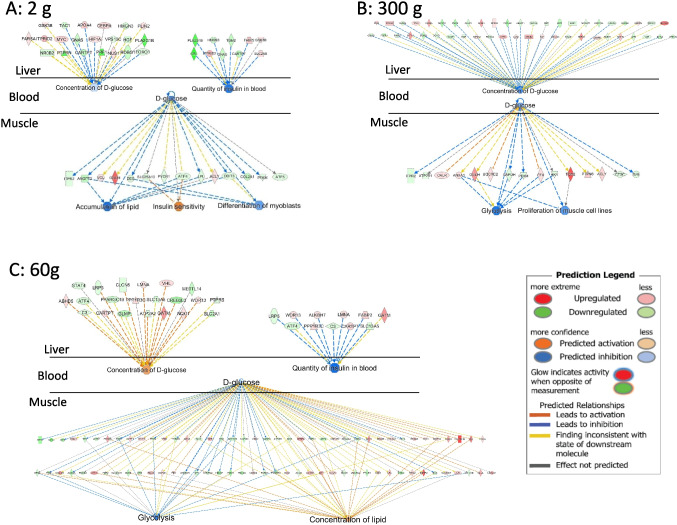
Network analysis describing how DEGs predict changes in hepatic concentration (and production) of glucose and insulin and thus affect expression of glucose-responsive DEGs and biological functions in skeletal muscle of the HY line. **A**) 2 g sampling, **B**) 300 g sampling, and **C**) 60 g sampling

In the muscle of the HY line (at all sampling periods), glucose was consistently identified as an inhibited upstream regulator (*P* < 0.05; 2 g, *z*-score =  − 1.276; 60 g, *z*-score =  − 0.486; 300 g, *z*-score =  − 0.853), potentially driving changes in the observed DEGs in the muscle (Fig. 9). In the 2 g and 300 g fish, this is likely due in part to reductions in hepatic glucose output as the “concentration of d-glucose” pathway was enriched in the liver (*P* < 0.05) with negative *z*-scores (2 g, *z*-score =  − 0.30; 300 g, *z*-score =  − 1.09) that reflect pathway inhibition (Fig. 9). In addition, at 2 g, the HY line also demonstrated enrichment of “quantity of insulin in blood” in an inhibitory manner, possibly contributing to regulation of glucose-responsive DEGs (*P* < 0.05, *z*-score =  − 1.57). However, in the 60 g fish, enrichment of the “concentration of d-glucose” pathway supported increased hepatic glucose production in the HY line (*P* < 0.05, *z*-score = 0.91), although a reduction in the quantity of insulin in blood (*P* < 0.05, *z*-score =  − 2.16) likely contributes to the DEGs for which glucose serves as a regulator.

### qPCR Validation

A subset of DEGs was selected for each tissue type (five genes per tissue) at three different body sizes to validate the transcriptomic analyses via qPCR. Those genes that overlapped body sizes and were relevant to growth and metabolic actions were selected when possible. The relative abundance of the genes measured was similar to the RNA-seq values, although in most cases the level of fold change was lower for qPCR expression with an average 0.43-fold change difference. The scatterplot for both muscle and liver genes shows a positive correlation with the linear regression between RNA-seq vs. qPCR expression data (Supplementary File 4; *R*^2^ = 0.7013, *P* < 0.001). More notably, the direction of regulation was in agreement for all genes measured, although several DEGs, when analyzed by qPCR, exhibited expression levels that were statistically similar (*P* > 0.05).

## Discussion

Fillet yield is one of the most economically valuable traits in fish farming. Selection for fillet yield for three generations is previously reported to increase carcass and fillet yield (percent and absolute yield) by 2–5 percentage points between the 500 g and 2 kg harvest weights, regardless of diet (Cleveland et al. 2024). Findings from the current study indicate that the high carcass yield (%) trait in the HY line appears at much earlier periods of development (60 g), supporting that physiological mechanisms directing the HY phenotype are active during early juvenile stages of growth. Condition factor is also greater in the HY line at 60 g, and this difference persists during grow-out to harvest weights (Cleveland et al. 2024).

There were 3663 DEGs identified between HY and LY lines of rainbow trout at three different body sizes (2 g, 60 g, 300 g) in two metabolically relevant tissues, the muscle and liver. These DEGs help to illustrate the physiological differences in rainbow trout genetically selected for high fillet yield and thus provide some understanding regarding genetic variation driving this phenotype. When specifically interrogating the DEGs for enrichment of biological pathways that direct muscle growth, findings suggested the increased muscle yield phenotype at 60 g partially results from higher rates of muscle cell proliferation, while muscle cell differentiation was downregulated, although later upregulated at 300 g. These findings are in agreement with a genome-wide association study in rainbow trout that determined genetic variation contributing to differences in the ability to maintain a proliferative population of myogenic precursor cells was associated with the fillet yield phenotype (Gonzalez-Pena et al. 2016).

Functionally significant DEGs potentially directing increased muscle cell proliferation rates in the HY line (60 g) include greater expression of *igf1*, *fgfr1*, *egfr*, *tgfb3*, and reduced expression of *myod1*. Insulin-like growth factor-1 (Igf1) is primarily recognized as a hepatic-derived endocrine hormone that is reported to upregulate muscle cell proliferation in primary myocyte cultures isolated from rainbow trout (Gabillard et al. 2010; Castillo et al. 2004). However, muscle-derived Igf1 is increasingly recognized for its physiological significance. A fivefold overexpression of *igf1* in the skeletal muscle of crucian carp enhanced myofiber hyperplasia and increased quantities of red muscle compared to white muscle, despite causing an overall loss in skeletal muscle mass (Li et al. 2014). Therefore, the 1.8-fold increase in *igf1* expression in muscle of the HY line likely promotes muscle cell hyperplasia, thereby contributing to greater muscle mass. Additionally, increased fibroblast growth factor (Fgf) signaling via upregulation of *fgfr1* expression in muscle may also stimulate muscle growth, as Fgf2 upregulates muscle cell proliferation in primary rainbow trout myocyte cultures (Gabillard et al. 2010). Increased expression of *tgfb3* is expected to promote muscle growth as well, as *tgfb3* is upregulated during post-fasting recovery growth, the expression of which is increased by treatment with Igf1 in vitro (de Mello et al. 2015). Little is known regarding the role of EGF on growth of muscle in fish, but in mammals this cytokine is well recognized for promotion of both proliferation and differentiation of myoblasts (Raab and Klagsbrun 1997). Knockout of the EGFR in zebrafish is lethal, but heterozygous fish exhibit greater numbers of slow-twitch muscle fibers (Ciano et al. 2019); therefore, the red to white muscle mass ratio (and thus fillet yield) may be skewed to the right when *egfr* is upregulated in rainbow trout. Furthermore, reduced expression of *myod*, a muscle-specific transcription factor that regulates myogenic specification and downstream cell differentiation factors, is expected to have a role in line-specific myogenic programs affecting myocyte differentiation and muscle growth.

In addition to hyperplasia, muscle growth is also driven by hypertrophy, the increase in cell size. The HY line exhibits a greater proportion of large-diameter muscle fibers at the 2-kg body weight (Cleveland et al. 2024), indicating that increased muscle yield is driven in part by hypertrophy. Central to hypertrophy is the accretion of protein, which requires protein synthesis rates to exceed protein degradation. Across all size ranges, DEGs in the HY line support that increased muscle fiber hypertrophy and greater rates of protein accretion are primarily attributed to a reduction in protein degradation rather than enhanced protein synthesis. In young fish (2–60 g), this appears to be driven by reduced autophagy, which is responsible for between 30 and 50% of total protein degradation in rainbow trout muscle cells in vitro (Seiliez et al. 2014). Although, a DEG downregulated in the HY line muscle at all sampling points was *glmp* (glycosylated lysosome membrane protein), encoding a protein that functions as a uniporter to shuttle dipeptides into and out of the lysosome (Jungnickel et al. 2024), suggesting that autophagy may be reduced in HY fish at all periods of development. Genetic variation in SNPs associated with *lamp2* (lysosome associated membrane protein 2), a protein with a similar function to GLMP, is also reported in rainbow trout with variation in fillet yield (Salem et al. 2018) and growth rates (Ali et al. 2020). Knockout of the *lamp2a* gene in rainbow trout increases growth rate and enhances muscle adiposity, confirming a functional role for chaperone-mediated autophagy in growth and nutrient partitioning in this species (Schnebert et al. 2024).

Higher rates of muscle growth in fish are typically associated with increased expression of collagen and other extracellular matrix-related genes that are essential for both the structure and function of this tissue (Harish et al. 2019; Yin et al. 2023; Zhao et al. 2021). Enrichment of DEGs associated with collagen synthesis/trimerization and extracellular matrix (ECM) organization was reported in skeletal muscle of fast-growing golden-line barbel (*Sinocyclocheilus grahami*) (Harish et al. 2019), zebrafish (*Dabio rerio*) (Yin et al. 2023), and large-scale loach (*Paramisgurnus dabryanus*) (Zhao et al. 2021). In the current study, components of the ECM exhibited size-specific patterns of expression. At the 2 g body size, all differentially expressed collagen transcripts in the HY line were downregulated, evidenced by negative *z*-scores of the enriched collagen-related pathways and the ECM organization pathway. At 60 g, the relationship was inverted, with positive *z*-scores for these pathways in the HY line. Enrichment of these terms reflects size-dependent growth trajectories, as the HY line exhibited slower growth at 2 g (12% lower body weight) and reduced capacity for both collagen biosynthesis/trimerization and ECM organization. In contrast, when muscle growth increased at the 60 g sampling (evidenced by higher % carcass yield), collagen and ECM-related genes were upregulated. Notable was the upregulation of the *col22a1* gene in the HY line at 60 g and 300 g; the collagen XXII protein is found in the basement membrane and localized at myotendinous junctions, the site of muscle force transmission (Vandeputte et al. 2019). Knockdown of *col22a1* in zebrafish induces a muscular dystrophy phenotype (Charvet et al. 2013), supporting an essential role for this protein for muscle development and/or function in fishes. Additionally, *hspg2*, the gene that encodes the perlecan protein, was upregulated in muscle tissue within the HY line at all sampling periods. Biological processes regulated by perlecan and other skeletal muscle proteoglycans include controlling vascularization, tissue regeneration, muscle growth, and differentiation (Velleman et al. 2012; Hayes et al. 2022) by promoting growth factor binding to cell receptors (Aviezer et al. 1994). Knocking out the *hspg2* gene in mice promoted fast-twitch muscle hypertrophy under mechanical overload but caused atrophy in slow-twitch muscle under mechanical unloading, supporting a role for perlecan in the muscle response to mechanical stimuli (Xu et al. 2010). Similarly, morpholino knockdown of *hspg2* in embryonic zebrafish elicited severe myopathy (Zoeller et al. 2008). Therefore, persistent elevated levels of *hspg2* expression in the HY line could be playing a role in the higher rates of muscle growth and development.

Interestingly, an overall negative regulation of glycolytic and gluconeogenic pathways was detected in the muscle of the HY line at 60 g, with a prominent 41% enrichment rate of DEGs within glycolysis (12 of 28 enzymes), most of which were downregulated. The three main rate-limiting enzymes in glycolysis are hexokinase (*hk*), phosphofructokinase (*pfk*), and pyruvate kinase (*pk*); two of these three enzymes exhibited reduced expression in muscle of the HY line at 60 g (m, muscle-specific *pfkm* and *pkm*) and 300 g (*hk* and *pfkm*), providing strong support for reduced capacity to breakdown glucose into energy substrates. Transcriptome analysis in other fish species also report enrichment of DEGs within the glycolytic pathway in muscle of faster growing fish, although the direction of regulation is inconsistent (Li et al. 2017, 2024; Wang et al. 2024; Lv et al. 2024; Xie et al. 2023; Yin et al. 2023; Lu et al. 2020; Sun et al. 2016). In the HY line, a reduced glycolytic capacity was somewhat unexpected, as DEGs suggested the HY line exhibit improved insulin sensitivity in muscle (60 g size), which should increase glucose uptake, the primary substrate for glycolysis. However, overexpression of skeletal muscle *igf1* in transgenic crucian carp (*Carassius auratus*) caused downregulation of *pfkma* and *pfkmb*, (Li et al. 2014), so greater expression of *igf1* in muscle of the HY line (60 g) may contribute to reduced expression of *pfkm*. Network analysis also revealed DEG expression patterns at all samplings that support reduced hepatic release of glucose and/or insulin into circulation, which is expected to affect expression of glucose-responsive genes, particularly reducing glycolytic gene expression. This analysis reveals potential interactive mechanisms linking physiological processes in the liver and muscle that collectively contribute to higher fillet yield in the HY line.

Reduced capacity for glycolysis in the HY line may reflect an increased reliance upon the more efficient TCA cycle for energy production. Supporting this concept is the notable upregulation of ATP-citrate synthase (*acly*) and oxoglutarate dehydrogenase (*ogdhb*) in the muscle of the high-yield line at all stages of development. ATP-citrate synthase (also known as citrate lyase) initiates the TCA cycle by producing citrate from acetyl-CoA and oxaloacetate. Interestingly, SNP markers associated with *acly* explained up to 8% of the genetic variance for the muscle yield in a population of rainbow trout; a 1.43-fold higher level of citrate synthase enzyme activity was also reported in rainbow trout with high muscle yield (Salem et al. 2018), providing strong support that this enzyme affects muscle mass. Three enzymatic steps later in the TCA cycle is oxoglutarate dehydrogenase, a key control point that catalyzes an oxidative decarboxylation step to produce the energy-rich substrates succinyl-CoA and NADH. The enzyme immediately upstream, NADH-producing isocitrate dehydrogenase (*idhg3*), and was also upregulated at 60 g. Therefore, the HY line appears to exhibit increased capacity for oxidative phosphorylation, potentially with increased reliance upon on acetyl-CoA derived from fatty acid oxidation rather than glycolysis. This efficient metabolic approach in the HY line may direct nutrient-dense metabolites toward support of muscle growth.

In addition to higher muscle yield, at 1.5–2 kg body weight the HY line exhibits approximately 20% reduced visceral fat content compared to the LY line (Cleveland et al. 2024). While this response may be due in part to greater utilization of lipids by muscle tissue, the liver also plays a significant role in nutrient partitioning. Expression patterns of DEGs in liver provide strong support that mechanisms of nutrient utilization differ between the HY and LY lines. Specific hepatic signaling pathways enriched with DEGs at all three sampling points include (1) regulation of lipid metabolism by PPARα, (2) insulin secretion signaling pathway, (3) protein kinase A signaling, (4) AMPK signaling, and (5) serotonin receptor signaling. While the direction of regulation of the initial four pathways depended on size, serotonin receptor signaling was consistently downregulated in the HY line and declined further with age (*z*-scores became progressively more negative). Metabolic effects of serotonin signaling are tissue dependent; in the brain, serotonin reduces appetite while in the liver it promotes gluconeogenesis (Park et al. 2021). Increased serotonin signaling also associates with diet-induced hepatic steatosis and lipid accumulation (Choi et al. 2018; Osawa et al. 2011), suggesting that a reduction in hepatic serotonin receptor signaling in the HY line contributes to reduced adiposity in these fish.

## Conclusion

Overall, we have identified numerous genes and pathways that are differentially regulated between HY and LY fish in both the liver and muscle, showing the basis of the physiological mechanisms that influence fillet yield phenotypes in rainbow trout. Regulation of the genes and pathways were not static and shifted over time based on the period of growth and developmental stage of the fish. Findings indicate that higher rates of muscle growth in the HY line are driven primarily by higher rates of protein accretion caused by reductions in protein degradation, increased muscle cell myogenesis (hyperplasia) around the 60 g size, potentially due to increased growth factor signaling, and greater muscle cell differentiation and hypertrophy in larger sizes. The high yield trait also appears to be accompanied by more efficient utilization of lipid oxidation products for energy in muscle, along with a hepatic-derived nutrient partitioning that reduces the visceral adiposity, indicating that mechanisms beyond those regulating muscle growth contribute to the high filet yield phenotype. These findings provide insight into the physiology behind genetic selection of the economically important trait of improved fillet yield and can contribute to development of markers to support genomic selection for growth performance traits.

## Supplementary Information

Below is the link to the electronic supplementary material.

ESM 1 (DOCX 17.6 KB)

ESM 2 (XLSX 19.6 KB)

ESM 3 (XLSX 1.26 MB)

ESM 4 (PPTX 59.5 KB)

## Data Availability

Raw sequence data is archived in NCBI as BioProject number PRJNA1242221 (https://www.ncbi.nlm.nih.gov/bioproject/).
