# Supplementary material for: Transcriptomic Profiles of Rainbow Trout (Oncorhynchus mykiss) Selectively Bred for High and Low Fillet Yield
Source: Mar Biotechnol (NY). 2025 Jun 25;27(4):102. doi: 10.1007/s10126-025-10479-0 (PMC12198287; doi:10.1007/s10126-025-10479-0)
Supplement: Supplementary file 1 — (DOCX 17.6 KB) [file 10126_2025_10479_MOESM1_ESM.docx]

Title: Transcriptomic Profiles of Rainbow Trout (Oncorhynchus mykiss) Selectively Bred for High and Low Fillet Yield

Journal: Marine Biotechnology

Authors: Jamie Mankiewicz, Guangtu Gao, Timothy Leeds, Beth Cleveland

Corresponding Author: Beth Cleveland, USDA/ARS/NCCCWA, beth.cleveland@usda.gov

Supplementary File 1. Primers used for validation of differentially expressed genes

| **Gene** | **Accession #** | **Direction** | **Primer (5'-3')** | **Product Size (bp)** | **Efficiency (%)** |
| --- | --- | --- | --- | --- | --- |
| *col15a1* | XM_021589371.2 | Forward | TCTCAGAGGTGCGGAAGGAC | 74 | 114 |
|  |  | Reverse | TGTGGCTAAGAGGTCCACCA |  |  |
| *col22a1* | XM_036970931.1 | Forward | GAGATCGGTGTACCTGGCG | 192 | 99 |
|  |  | Reverse | CCCTTTGGATCCTTCATCGC |  |  |
| *fabp2* | XM_021619074.2 | Forward | AGAGGAACTTGTACAGAGCTACAG | 202 | 98 |
|  |  | Reverse | CAGCTAAAAATGATGTTGGCGT |  |  |
| *gapdh* | XM_021623341.2 | Forward | TCCCCACTCACACCCGAAAG | 70 | 101 |
|  |  | Reverse | CTGAGTTGGTCTAGTCCTCGTT |  |  |
| *hspg2* | XM_036946987.1 | Forward | TCCGCAACGTGTTGTATGGA | 109 | 104 |
|  |  | Reverse | TTCACAAGGGGCAGAGCTTC |  |  |
| *igf-1* | XM_021577176.2 | Forward | GAGGCGGCTCGAAATGTACT | 196 | 95 |
|  |  | Reverse | ATGCCCAGATAAAGGTTTCTTCTT |  |  |
| *insr2* | XM_021599800.2 | Forward | TGGATATCTTCAAAACACACAGAGA | 137 | 99 |
|  |  | Reverse | TCCTTACTCTGACAGATTTCTCCA |  |  |
| *mfap4* | XM_021557923.2 | Forward | ATGCCAGTGGATTGTGCTGA | 164 | 99 |
|  |  | Reverse | TCCTTTCTCTTCTGGATCACTGTC |  |  |
| *pebp1* | XM_021619877.2 | Forward | CAGCTCCTGTTTGCAGCTCT | 181 | 101 |
|  |  | Reverse | CGGCTCTGCACCTGTGTT |  |  |
| *pfkmb* | XM_021569297.2 | Forward | TCTGGTCACAGGCAGTTCCA | 194 | 100 |
|  |  | Reverse | CCAAACCCTGGTAACCCTCAT |  |  |

*hspg2*: heparan sulfate proteoglycan 2; *pfkmb*: phosphofructokinase, muscle; *col22a1*: collagen type XXII alpha 1 chain; *gapdh*: glyceraldehyde-3-phosphate dehydrogenase; *igf-1*: insulin-like growth factor 1; *mfap4*: microfibril associated protein 4; *fabp2*: fatty acid binding protein 2; *col18a1*: collagen type XV alpha 1 chain; *pebp1*: phosphatidylethanolamine binding protein 1; *insr2*: insulin receptor 2
