# Supplementary material for: Transcriptomic Profiles of Rainbow Trout (Oncorhynchus mykiss) Selectively Bred for High and Low Fillet Yield
Source: Mar Biotechnol (NY). 2025 Jun 25;27(4):102. doi: 10.1007/s10126-025-10479-0 (PMC12198287; doi:10.1007/s10126-025-10479-0)
Supplement: Supplementary file 4 — (PPTX 59.5 KB) [file 10126_2025_10479_MOESM4_ESM.pptx]

## Slide 1
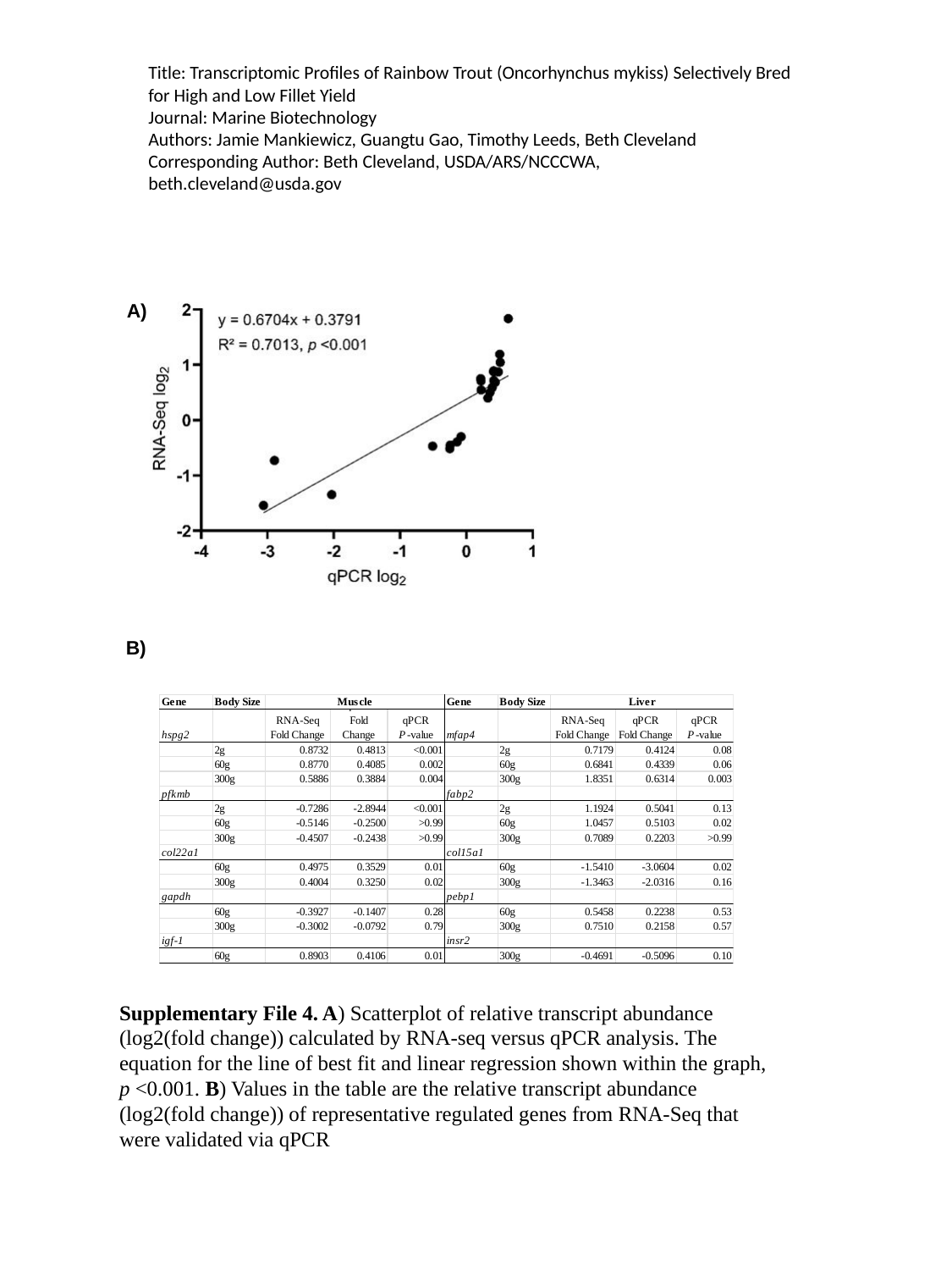

Title: Transcriptomic Profiles of Rainbow Trout (Oncorhynchus mykiss) Selectively Bred for High and Low Fillet Yield
Journal: Marine Biotechnology
Authors: Jamie Mankiewicz, Guangtu Gao, Timothy Leeds, Beth Cleveland
Corresponding Author: Beth Cleveland, USDA/ARS/NCCCWA, beth.cleveland@usda.gov
A)
B)
Supplementary File 4. A) Scatterplot of relative transcript abundance (log2(fold change)) calculated by RNA-seq versus qPCR analysis. The equation for the line of best fit and linear regression shown within the graph, p <0.001. B) Values in the table are the relative transcript abundance (log2(fold change)) of representative regulated genes from RNA-Seq that were validated via qPCR
